# The intriguing modeling of *cis–trans s*electivity in ruthenium-catalyzed olefin metathesis

**DOI:** 10.3762/bjoc.7.7

**Published:** 2011-01-11

**Authors:** Naeimeh Bahri-Laleh, Raffaele Credendino, Luigi Cavallo

**Affiliations:** 1 MoLNaC - Modeling Lab for Nanostructures and Catalysis (http://www.molnac.unisa.it), Dipartimento di Chimica, Università di Salerno, Via Ponte don Melillo, I-84084 Fisciano (SA), Italy; 2 Polymerization Engineering Department, Iran Polymer and Petrochemical Institute (IPPI), P.O. Box 14965/115, Tehran, Iran

**Keywords:** *cis–trans* selectivity, cross metathesis, DFT calculations, olefin metathesis, Ru-catalyst

## Abstract

In this study we have investigated computationally the origin of the *cis*–*trans* selectivity in the Ru-catalyzed cross metathesis (CM) of a prototype monosubstituted olefin, i.e., propene. Our calculations suggest that the origin of the preferential formation of *trans-*olefins is in the product release step, which prevents the initially formed *cis-*olefin from escaping the metal, and returns it to the reaction pool until the *trans-*olefin is formed.

## Introduction

**Scheme 1:**
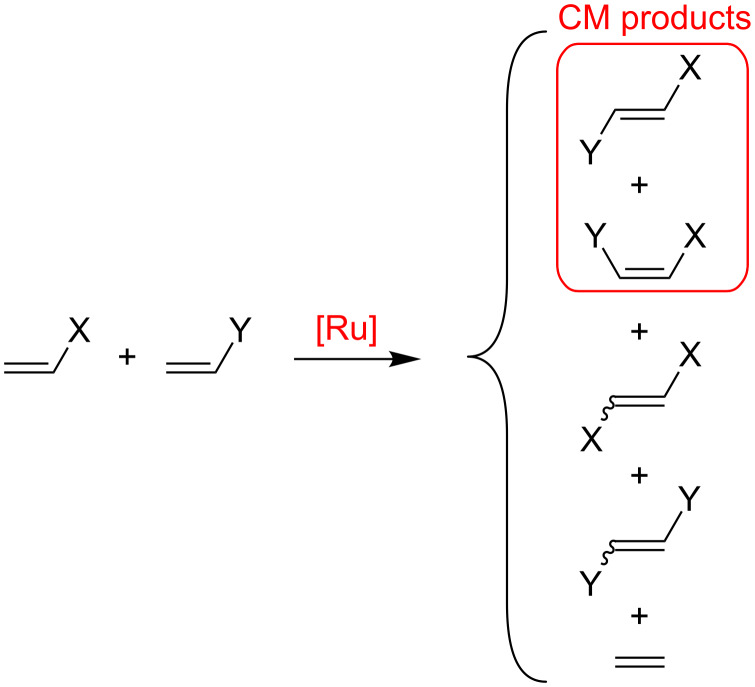
Possible products resulting from the CM of terminal olefins.

Olefin metathesis is among the most versatile tools when C=C double bonds must be manipulated. For this reaction Ru-based catalysts of various generations are particularly attractive due to their high tolerance for other functional groups [1–4]. Among the most useful possibilities is cross metathesis (CM), see Scheme 1, since it opens the door to the formation of functionalized and/or higher olefins from simpler unsaturated building blocks. Such a wide potential explains why CM applications span from the production of raw materials [5–6] to advanced and challenging organic synthesis [7–10]. On the other hand, the general scope of CM has made a challenging task the comprehension of the working mechanism and the development of rules to control this powerful synthetic tool [11–18]. Among the problems connected with the CM of reactive monosubstituted olefins are the minimization of homodimers and a control over the *cis–trans* selectivity, see Scheme 1. While minimization of homodimers can be achieved with proper handling of the reaction protocol [15], controlling the *cis–trans* selectivity is much more complicated, and is usually biased towards the formation of the *trans-*isomer.

**Scheme 2:**
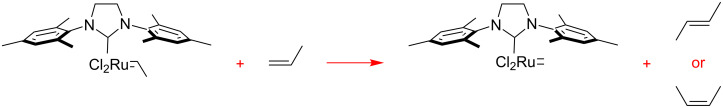
Representation of the reactions investigated.

For this reason, we decided to investigate computationally the *cis–trans* selectivity in the CM of the simplest terminal olefin, i.e., propene, to yield either *cis-* and *trans-*2-butene with the well characterized 2^nd^ generation Ru-catalyst based on the SIMes *N*-heterocyclic carbene ligand, see Scheme 2. Although it is well known that the steric hindrance of the olefin substituent has a remarkable role on both reactivity and products distribution, propene can still be considered as a prototype of terminal olefins, and can provide insights into the energetics of the basic CM reaction. Steric or electronic effects that would arise from more complex olefins would add to the prototype energy profile investigated here. Finally, for the sake of brevity, we will focus only on the productive CM of propene with the Ru–propylidene bond, while degenerate propene metathesis with the Ru–propylidene moiety, or propene reactivity with the Ru–methylidene moiety, which would be the other Ru–alkylidene bonds present in the reaction mixture after complete activation of the starting precatalyst, are not considered.

## Results and Discussion

**Figure 1:**
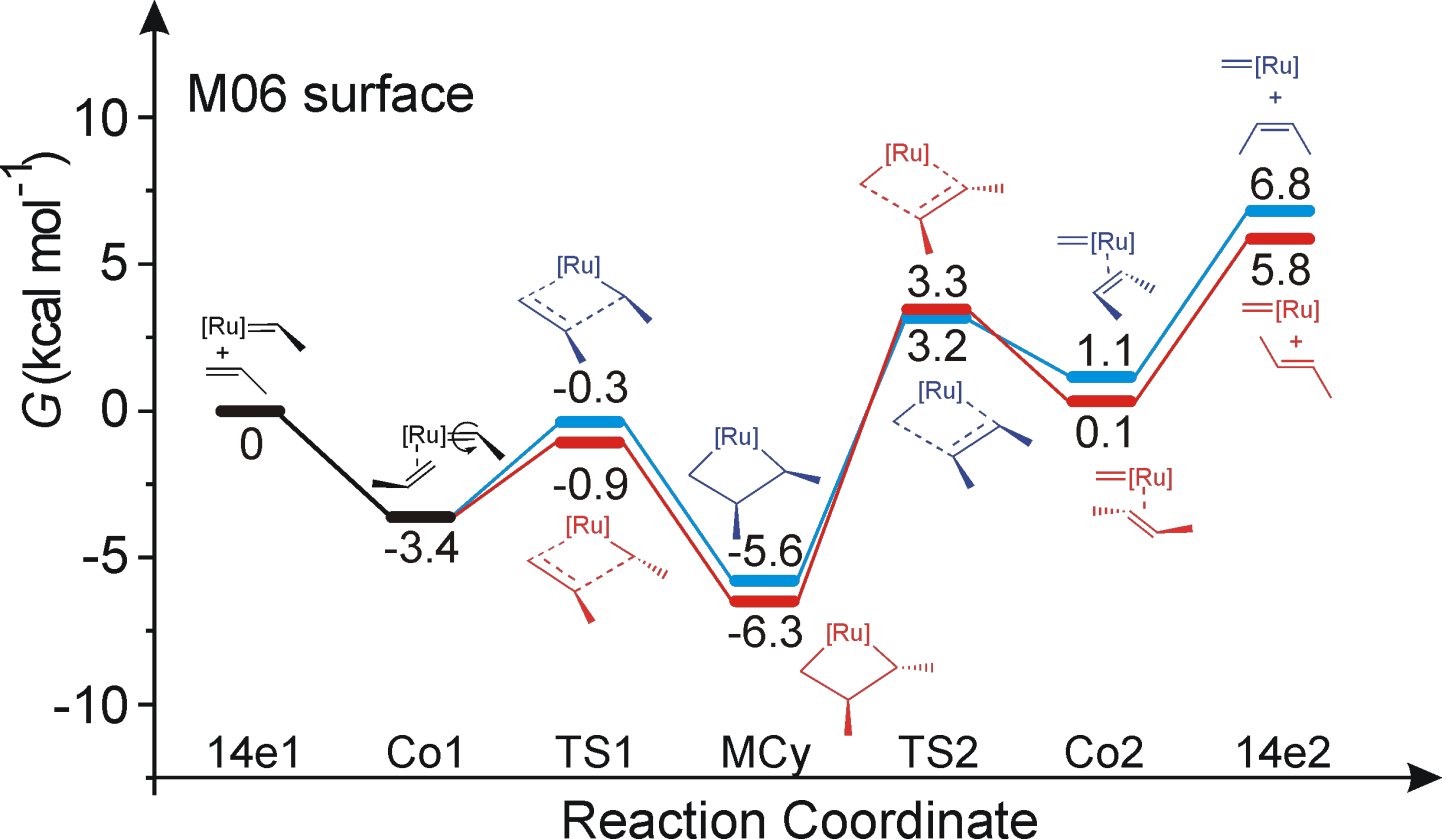
Reaction profile for the formation of *cis-* and *trans-*2-butene.

The free energy profile in CH_2_Cl_2_ for the formation of both *cis-* and *trans-*2-butene from the starting 14e Ru–propylidene species is shown in Figure 1. Coordination of propene to the Ru center of **14e1**, leading to the coordination intermediate **Co1**, is favored by 3.4 kcal·mol^−1^. In this intermediate the C=C double bond of the coordinated propene molecule is roughly perpendicular to the Ru–propylidene bond. From intermediate **Co1**, two transition states corresponding to productive metathesis, **TS1-*cis*** and **TS1-*trans***, can be reached via rather low energy barriers (3.1 and 2.5 kcal·mol^−1^ for **TS1-*cis*** and **TS1-*trans***, respectively). The two transition states differ in the relative orientation of the Me groups of the propene group and of the propylidene moiety, *syn* in **TS1-*cis***, and *anti* in **TS1-*trans***, with **TS1-*trans*** favored by 0.6 kcal/mol The experimentally proved free and fast rotation around the Ru–alkylidene bond allows both **TS1-*cis*** and **TS1-*trans*** to be reached from **Co1** [19–20]. The two transition states collapse into the corresponding **MCy-*cis*** and **MCy-*trans*** metallacycles, at 5.6 and 6.3 kcal·mol^−1^ below the starting **14e1** intermediate, respectively. The two metallacycles are the most stable structures along the reaction pathway. Release of *cis-* and *trans-*butene requires the breaking of the **MCy-*cis*** and **MCy-*trans*** metallacycles through the transition states **TS2-*cis*** and **TS2-*trans***, respectively, with formation of the two coordination intermediates **Co2-*cis*** and **Co2-*trans***, from which *cis-* and *trans-*butene are released. In this case, breaking the metallacycles is energetically quite expensive, around 9–10 kcal·mol^−1^, and transition states **TS2-*cis*** and **TS2-*trans***, in agreement with other computational studies, are higher in energy than the transition states for metallacycle formation **TS1-*cis*** and **TS1-*trans*** [21]. The **Co2-*cis*** and **Co2-*trans*** coordination intermediates are slightly more stable than the corresponding **TS2-*cis*** and **TS2-*trans*** transition states, which means that the energy barrier for the backward reaction that would return the **Co2-*cis*** and **Co2-*trans*** coordination intermediates back into the corresponding metallacycle is rather low (2.1 and 3.2 kcal·mol^−1^ for **Co2-*cis*** and **Co2-*trans***, respectively).

This scenario indicates a rather surprising and unexpected result. First, *cis* versus *trans* selectivity is not determined at metallacycle formation, since both the *cis* and *trans* TS1 transition states are roughly 3–4 kcal·mol^−1^ lower in energy that the TS2 transition states. Second, neither is selectivity determined at metallacycle breaking. In fact, the **TS2-*cis*** and **TS2-*trans*** transition states, within the accuracy of this type of calculation, are of the same energy. On the other hand, and in line with expectations, the **MCy-*trans*** metallacycle is more stable than the **MCy-*cis*** metallacycle by roughly 1 kcal·mol^−1^, and the **Co2-*trans*** coordination intermediate is similarly more stable than the **Co2-*cis*** coordination intermediate. Finally, our computational approach also reproduces well the experimental higher stability, by 1.0 kcal·mol^−1^, of *trans-*2-butene relative to *cis-*2-butene in the gas-phase [22].

To test whether the relative stability of the four transition states could depend on the chosen HMGGA M06 functional [23], we localized the transition states for metallacycle formation and breaking with the popular GGA BP86 [24–26] and HGGA B3LYP [27–29] functionals, as well as with the more recent MGGA M06L functional [30]. All these tests are summarized in Table 1. First, in all the cases **TS1-*cis*** is somewhat higher in energy than **TS1-*trans*** but, more importantly, both the TS1 metallacycle forming transition states are 3–5 kcal·mol^−1^ lower in energy than the TS2 metallacycle breaking transition states. Considering that all the functionals we considered indicate that the metallacycle forming transition state is not rate (selectivity) determining, we will not discuss this point any further.

**Table 1:** Energy difference, in kcal·mol^-1^, between the *cis* and *trans* transition states **TS1** and **TS2** of Figure 1, calculated with different computational approaches. In all cases **TS1-*trans*** is taken as reference at 0 kcal·mol**^−1^**.

Functional	Transition State	Δ*E*^≠^*_cis_*_–_*_trans_* gas-phase	Δ*G*^≠^*_cis_*_–_*_trans_* gas-phase	Δ*G*^≠^*_cis_*_–_*_trans_* CH_2_Cl_2_

BP86	**TS1-*trans***	0.0	0.0	0.0
	**TS1-*cis***	0.2	1.2	1.4
	**TS2-*trans***	4.5	4.1	4.1
	**TS2-*cis***	4.1	3.8	3.8

B3LYP	**TS1-*trans***	0.0	0.0	0.0
	**TS1-*cis***	0.5	0.1	0.3
	**TS2-*trans***	4.2	3.3	3.2
	**TS2-*cis***	3.7	2.1	2.0

M06L	**TS1-*trans***	0.0	0.0	0.0
	**TS1-*cis***	0.1	2.3	2.2
	**TS2-*trans***	6.0	6.5	5.6
	**TS2-*cis***	4.6	5.7	5.4

M06	**TS1-*trans***	0.0	0.0	0.0
	**TS1-*cis***	0.6	0.6	0.5
	**TS2-*trans***	4.9	4.6	4.2
	**TS2-*cis***	4.3	4.6	4.1

Focusing on the metallacycle breaking transition state, the values in Table 1 indicate that the difference between the **TS2-*cis*** and **TS2-*trans*** transition states is somewhat dependent on the computational approach used. Indeed, the BP86, B3LYP and M06L results suggest that the **TS2-*cis*** transition state is somewhat preferred over the **TS2-*trans*** transition state, thus suggesting a small preference for the formation of *cis-*olefins. The M06 functional, instead, indicates that these two transition states are practically of the same energy. Further, the values given in Table 1 indicate that in terms of gas-phase potential energy the **TS2-*cis*** is reasonably favored over **TS2-*trans*** also with the M06 functional. It is the inclusion of the vibrational/entropic part that makes the M06 *cis-* and *trans* transition states of similar energy, (compare the Δ*E*^≠^*_cis_*_–_*_trans_* and Δ*G*^≠^*_cis_*_–_*_trans_* values in the gas-phase in Table 1). Inclusion of solvent effects does not change the gas-phase trends (compare the Δ*G*^≠^*_cis_*_–_*_trans_* in gas-phase and the Δ*G*^≠^*_cis_*_–_*_trans_* in CH_2_Cl_2_ values in Table 1). In conclusion, if any preference exists at the level of the metallacycle breaking step, the consensus emerging from the comparison of various functionals is that the *cis* transition state is favored. The fact that the *cis* transition state is of lower or similar energy to the *trans* transition state, despite of the higher stability of the forming *trans* C=C skeleton, indicates that the SIMes ligand is more suitable to host a *cis* forming C=C bond rather than a *trans* C=C bond.

Based on these calculations, the conclusion emerging from the energy profiles of Figure 1 and the values given in Table 1 is that, in the framework of a dissociative mechanism, the key step determining the experimentally observed preferential formation of *trans-*olefins is product release with formation of the methylidene Ru 14e intermediate **14e2** and a free *cis-* or *trans-*2-butene molecule from **Co2-*cis*** and **Co2-*trans*** (in this regard it must be noted that an associative mechanism has been proposed to be operative for ethylene degenerate metathesis at low temperature [31–32], and for the activation step in Hoveyda/Grela type catalysts [33]). Product release is endoergonic due to the coordination energy of 2-butene that amounts to roughly 6 kcal·mol^−1^ both for the *cis-* and *trans-*isomers, and is clearly higher in energy than both TS2 transition states that would revert the just formed 2-butenes into the most stable metallacycle. For this reason, the energy profile of Figure 1 suggests that the most likely event from **Co2-*cis*** and **Co2-*trans*** is not product release, but rather their transformation into **MCy2-*cis*** and **MCy2-*trans***. The escape from **Co2-*cis*** and **Co2-*trans*** is controlled by the free energy difference between **14e2** and transition states **TS2-*cis*** and **TS2-*trans***, which amounts to 3.6 and 2.5 kcal·mol^−1^ for the *cis* and *trans* pathways, respectively. These numbers indicate that release of *trans-*2-butene is favored by the higher stability of the *trans-*olefin relative to the *cis-*isomer*.*

Of course, considering the high reactivity of both *trans-* and *cis-*butene towards metathesis, it is clear that secondary metathesis of the produced butenes, whose energetic can be still derived from Figure 1, will result in a statistical distribution of the products according to their thermodynamic stability [15,18]. Thus, a high *trans*/*cis-*butene ratio would be reached, even if a lower *trans*/*cis* ratio was initially produced. This conclusion is in qualitative agreement with CM experiments using terminal olefins, which resulted in a *trans/cis* ratio of around 2–4 at low conversions, that increased to 9–10 at higher conversions [18]. Finally, it is clear that the steric and electronic properties of more complex olefins can have a strong impact on the energy profile of Figure 1, which however, remains the base energy profile to be modified. We are currently working in this direction.

## Conclusion

In this study we have investigated computationally the origin of the *cis–trans* selectivity in the CM of the prototype monosubstituted olefin, i.e., propene. Our calculations suggest that the origin of the preferential formation of *trans-*olefins is not the energy difference at the transition states corresponding to either metallacycle formation of breaking. Actually, focusing on the transition state higher in energy (the one corresponding to metallacycle breaking), we found that the transition state leading to the formation of *cis-*butene is of similar energy, or even favored, relative to that leading to the formation of *trans-*butene. Thus, CM of propene (and by consequence of simple linear 1-olefins) should kinetically lead first to the formation of *cis*-olefins, followed by gradual conversion to the more stable *trans-*isomer. Our calculations suggest that the key step to rationalize the preferential formation of *trans-*olefins, even at low conversion, is in the product release step, since *trans*-olefins have a higher tendency to be released from the catalyst at the end of the CM reaction. Conversely, the initially formed *cis*-olefins have a minor tendency to be released from the catalyst, and thus they have a higher chance to return to the reaction pool until the *trans-*olefin is formed.

## Experimental

### Computational Details

The DFT calculations of the full energy profile of Figure 1 were performed at the HMGGA level with the Gaussian09 package [34], using the M06 functional of Truhlar [23]. Free energies in CH_2_Cl_2_ were deduced from the gas-phase free energies plus the solvation energy term estimated in single point calculations on the gas-phase optimized structures, based on the polarizable continuum solvation model PCM using CH_2_Cl_2_ as the solvent [35]. In case of olefin coordination, we assumed a –*T*Δ*S* contribution of 10 kcal·mol^−1^, since the gas-phase rotational/translational entropy of coordination from classical statistical thermodynamics is generally considered to overestimate the coordination entropy in solution. The –*T*Δ*S* contribution of 10 kcal·mol^−1^ is the experimental coordination entropy of C_2_H_4_ to a Pd-complex [36]. The test calculations in Table 1 were performed with the GGA BP86 functional of Becke and Perdew [24–26], with the HGGA B3LYP functional of Becke, Lee, Parr, and Yang [27–29], and with the MGGA M06L functional of Truhlar [30]. In all cases the electronic configuration of the molecular systems was described with the standard split-valence basis set with a polarization function of Ahlrichs and co-workers for H, C, N, O and Cl (SVP keyword in Gaussian03) [37]. For Ru we used the small-core, quasi-relativistic Stuttgart/Dresden effective core potential, with an associated (8s7p6d)/[6s5p3d] valence basis set (SDD keywords in Gaussian03) [38]. Characterization of the located stationary points as minima or transition state was performed by frequency calculations.
